# Bacteriophages with depolymerase activity in the control of antibiotic resistant *Klebsiella pneumoniae* biofilms

**DOI:** 10.1038/s41598-023-42505-3

**Published:** 2023-09-13

**Authors:** Fedor Zurabov, Egor Glazunov, Tatiana Kochetova, Viktoria Uskevich, Valentina Popova

**Affiliations:** 1 Research and Production Center “MicroMir”, LLC, Moscow, Russia; 2 Department of Virology, Lomonosov Moscow State University, Moscow, Russia

**Keywords:** Bacteria, Bacteriophages, Biofilms

## Abstract

*Klebsiella pneumoniae* is associated with a variety of infections, such as pneumonia, urogenital infection, liver abscess, and bloodstream infection. It is especially dangerous for patients in medical facilities, where it can cause ventilator-associated pneumonia or intensive care unit-acquired pneumonia. The emergence of multidrug-resistant and hypervirulent strains as well as the ability to form biofilms on various medical devices complicates the treatment of such infections and makes the use of antibiotics ineffective. The application of bacteriophages is a promising alternative for combating *Klebsiella pneumoniae* biofilms. In the present study a cocktail of 3 bacteriophages with depolymerase activity was used to control antibiotic resistant *Klebsiella pneumoniae* biofilms in vitro. Biofilms were examined using optical and scanning electron microscopy. The obtained results demonstrate that the studied bacteriophage cocktail can effectively disrupt *Klebsiella pneumoniae* biofilms.

## Introduction

*Klebsiella pneumoniae* is a Gram-negative bacterium associated with different human health conditions such as pneumonia, bloodstream infection, urogenital infection, etc. It is one of the most frequently detected bacteria in human respiratory tract infections, especially in healthcare patients, as it is often linked to ventilator-associated pneumonia (VAP) and intensive care unit (ICU)-acquired pneumonia^1^, which are among the most common bacterial complications following viral respiratory tract infections, including severe acute respiratory syndrome-related coronavirus 2 (Sars-Cov-2)^2,3^. It has been reported that pan drug resistant (PDR) *K. pneumoniae* is the most frequently isolated bacteria in critically ill coronavirus disease 2019 (COVID-19) patients under mechanical ventilation^4^. The treatment of such infections is complicated by increasing levels of resistance to antimicrobial drugs. An increase in the incidence of *K. pneumoniae* infections caused by carbapenem-resistant strains has been detected worldwide, which has resulted from the use of carbapenem-class antibiotics against extended spectrum β-lactamases (ESBL)-producing strains of *K. pneumoniae*^5–7^. Global studies show that a significant proportion of nosocomial *K. pneumoniae* isolates exhibit extended spectrum β-lactamases (ESBLs) and carbapenemases activity^8^. Some *K. pneumoniae* isolates are resistant to the most of clinical antimicrobial agents (pan drug resistant), such as aminoglycoside, quinolone, β-lactam antibiotics, polymyxin and tigecycline^9,10^.

*K. pneumoniae* strains are able to form biofilms, which are extracellular polymeric structures consisting of deoxyribonucleic acid (DNA), proteins, and polysaccharides. *K. pneumoniae* biofilm growth includes the adhesion of cells, the development of small clusters, the maturation, and the release of free-living planktonic cells^11^. Biofilm development on catheters, tubes and other medical equipment increases the risk of acute infections in patients receiving medical care, especially in those on prolonged mechanical life support, because the biofilm matrix not only physically protects the bacterial cells, but also contributes to the transmission of mobile genetic elements, which increases the tolerance of microorganisms to antibiotics^12^.

The emergence of antibiotic resistance and the reduced possibility of treating the sick patients encourages researchers to pursue novel approaches to regulating bacterial growth and re-establishing the microbiota's natural equilibrium. Bacteriophages are increasingly being used by scientists and clinicians to control antibiotic resistant strains of bacteria^13^. The use of bacteriophages against antibiotic resistant bacteria has been shown to be successful in limiting bacterial growth, as well as restoring susceptibility to antimicrobial drugs^14^. The described effects are probably associated with a switch in the adaptation mechanisms of the bacterial population to bacteriophage infection. Furthermore, bacteriophages whose genomes encode exopolysaccharide depolymerases can use exopolysaccharides as primary receptors and cleave polymer bonds until reaching the cell membrane, which contributes to the biofilm degradation and infection of resident bacteria^15,16^.

The aim of the present study was to test the efficacy of the previously characterized bacteriophages encoding polysaccharide depolymerases against *K. pneumoniae* biofilms.

## Material and methods

### Bacteriophages

Bacteriophages vB_KpnS_FZ10, vB_KpnP_FZ12 and vB_KpnM_FZ14 that had been previously isolated from sewage waters and fully characterized^17^ were used in this study.

The selected bacteriophages were tested in vivo as a part of a broader phage cocktails in published studies^18,19^. Real-time in vitro analysis of *K.pneumoniae* (Kl 315) culture lysis with a cocktail of selected phages was carried out in a previous study using imaging with a 3D Cell Explorer microscope^19^.

### Bacterial strains

For the bacteriophage efficiency study, the multidrug-resistant clinical strain Kl 315 of *K. pneumoniae* from the RPC “MicroMir” collection was selected. The strain was obtained from a patient with pneumonia, examined on a MALDI-TOF Microflex mass spectrometer (Bruker, Billerica, MA, USA) and with biochemical tests (MIKROLATEST, Erba Mannheim) with further analysis on Multiskan Ascent spectrophotometer (Thermo Scientific, Waltham, MA, USA) and was tested for antibiotic susceptibility by a disc diffusion test (Kirby Bauer method) for antimicrobials using the European Committee on Antimicrobial Susceptibility Testing (EUCAST) and Clinical Laboratories Standards Institute (CLSI) standards before adding to collection. Antibiotic susceptibility test results are presented in Supplementary Table S1. The same strain was chosen to examine the properties of the bacteriophages used in this study^17^.

### Biofilm formation

For biofilm formation, 18-h cultures of Kl 315 strain grown on Brain Heart Infusion (BHI) agar (Himedia, India) were used. Suspension of bacteria at a concentration of 1 × 10^5^ CFU/ml in a volume of 5 ml was shaken on a Vortex mixer (Misrospin FV-2400, Biosan, Latvia) and added to Petri dishes with 20 ml of BHI broth (Himedia, India). Sterile glass slides were placed on the bottom of Petri dishes, then sterile degreased coverslips were placed on top of the slides and incubated in the thermostat (Binder GmbH, Germany) for 24–72 h at 37 °C.

### Biofilm infection with bacteriophages

After 24 h of Kl 315 biofilm formation, 0.1 ml of bacteriophage cocktail with a concentration of 1 × 10^7^ PFU/ml of each phage (vB_KpnS_FZ10, vB_KpnP_FZ12 and vB_KpnM_FZ14) or a single phage vB_KpnP_FZ12 was added and incubated in the thermostat (Binder GmbH, Germany) for 24–48 h at 37 °C. Control glass slides with glass coverslips were incubated without bacteriophage addition.

### Biofilm fixation

For optical microscope visualization, coverslips were removed with tweezers and placed into Petri dishes with paper filters at the bottom without drying. To preserve the natural form of biofilms, samples were vapor-fixed with 25% glutaric aldehyde for 3 h. After fixation, the samples were stained with DAPI (Sigma-Aldrich, Germany). The effect of bacteriophage cocktail on biofilm formation and destruction was evaluated using an AxioImager A1 light microscope (Carl Zeiss, Germany).

For scanning electron microscopy (SEM), samples were fixed with 2.5% glutaraldehyde for 60 min, then dehydrated in a graded ethanol series (30, 50, 70, 80, and 96%) and placed into acetone. The samples were dried in a critical-point dryer HCP-2 (Hitachi Ltd., Japan) and coated with Au–Pd in IB-3 ion coater (Eiko Engineering Co., Tokyo, Japan). Samples were visualized in Camscan-S2 (Cambridge, United Kingdom) scanning electron microscope.

## Results

### Bacteriophages

In a previous study^17^, the morphology of the bacteriophages vB_KpnS_FZ10, vB_KpnP_FZ12 and vB_KpnM_FZ14 was evaluated by transmission electron microscopy, thermal and pH stability were assessed, one-step growth parameters, host adsorption rate, host range and phage resistant form generation frequency were characterized, and full genome sequencing and analysis were performed. All 3 bacteriophages encode polysaccharide depolymerases and form plaques with halo. A cocktail of 3 phages was selected for further in vitro and in vivo studies, as the evaluation of lytic spectra and the frequency of generation of phage-resistant forms showed that the use of the selected phage cocktail not only increases the lytic efficacy of the preparation, but also significantly reduces the risk of formation of phage-resistant forms. The tail fiber proteins of all 3 phages differed in their folding and domain structure, as was found by analyzing homologues of structural proteins from the UniProt database (vB_KpnS_FZ10–A0A4D6T3L6; vB_KpnP_FZ12–A0A4D6T3P7; vB_KpnM_FZ14–A0A4D8SZG4; vB_KpnS_FZ41–A0A4D6T3Y8). Tail proteins of bacteriophages vB_KpnS_FZ10, vB_KpnP_FZ12 and vB_KpnM_FZ14 were shown to share homology to phage proteins with known endosialidase, peptidoglycan hydrolase, and hyaluronate lyase domains, respectively, all of which have been confirmed to have polysaccharide degrading activity. The titers obtained by growing the phages in liquid nutrient medium showed that they could effectively inhibit the growth of *K. pneumoniae* culture and indicated sufficient virus productivity to obtain high concentrations in the final preparation. A real-time in vitro phage lysis assay showed that the selected phage cocktail could effectively lyse Kl 315 planktonic cells of *K. pneumoniae* culture and disrupt small microcolonies adhered on the glass surface^19^. Therefore, the cocktail of bacteriophages vB_KpnS_FZ10, vB_KpnP_FZ12 and vB_KpnM_FZ14 was chosen for further studies on more mature biofilms carried out in the present work and vB_KpnP_FZ12 was selected as the most productive on Kl 315 strain to compare the efficacy of a single phage vs three-phage cocktail on *K. pneumoniae* biofilms.

### Optical microscope biofilm imaging

**Figure 1:**
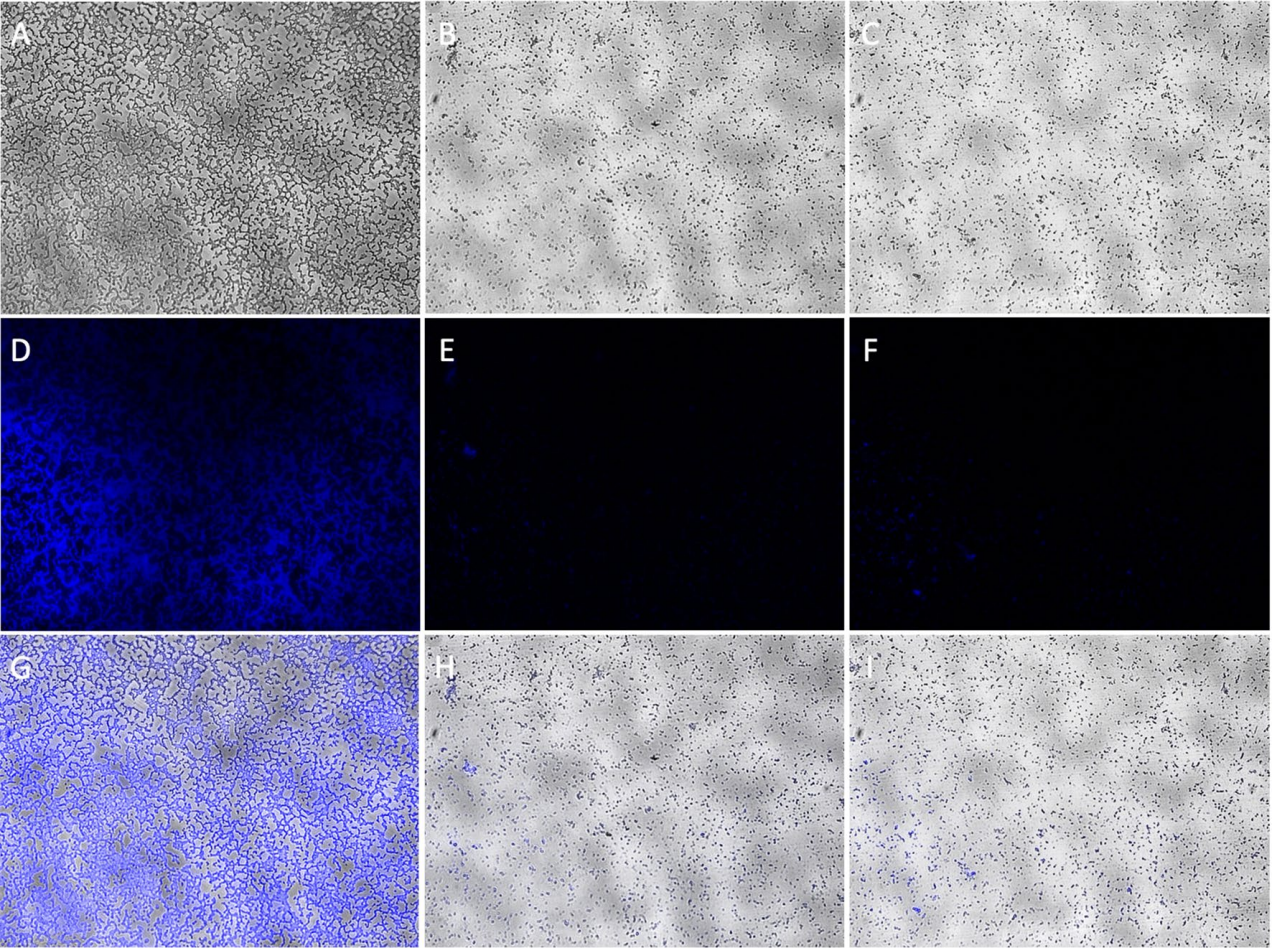
AxioImager A1 light microscope micrographs of *K. pneumoniae* (Kl 315) culture: (**A**,**D**,**G**) after 48 h of incubation without phages; (**B**,**E**,**H**) 24 h of incubation + 24 h of incubation after vB_KpnP_FZ12 addition; (**C**,**F**,**I**) 24 h of incubation + 24 h of incubation after bacteriophage cocktail addition; (**A**–**C**) transmitted light micrographs; (**D**–**F**) DAPI fluorescence micrographs; (**G**–**I**) transmitted light + DAPI fluorescence combined micrographs. Magnification × 400. Experiment was conducted in at least three repetitions; representative slides were presented.

After 24 h of Kl 315 biofilm formation, a single phage vB_KpnP_FZ12 or a cocktail of bacteriophages vB_KpnS_FZ10, vB_KpnP_FZ12 and vB_KpnM_FZ14 was added to the biofilm and incubated for additional 24 h. Control glasses were incubated for 48 and 72 h without the addition of bacteriophages. After 48 h of incubation the formation of chains and clusters was observed in control glasses, which is common for biofilms of all bacterial species, while only single cells and small colonies were observed on the experimental glasses with the addition of phages (Fig. 1). The antibiofilm activity of the phage cocktail was similar to that of single vB_KpnP_FZ12.

**Figure 2:**
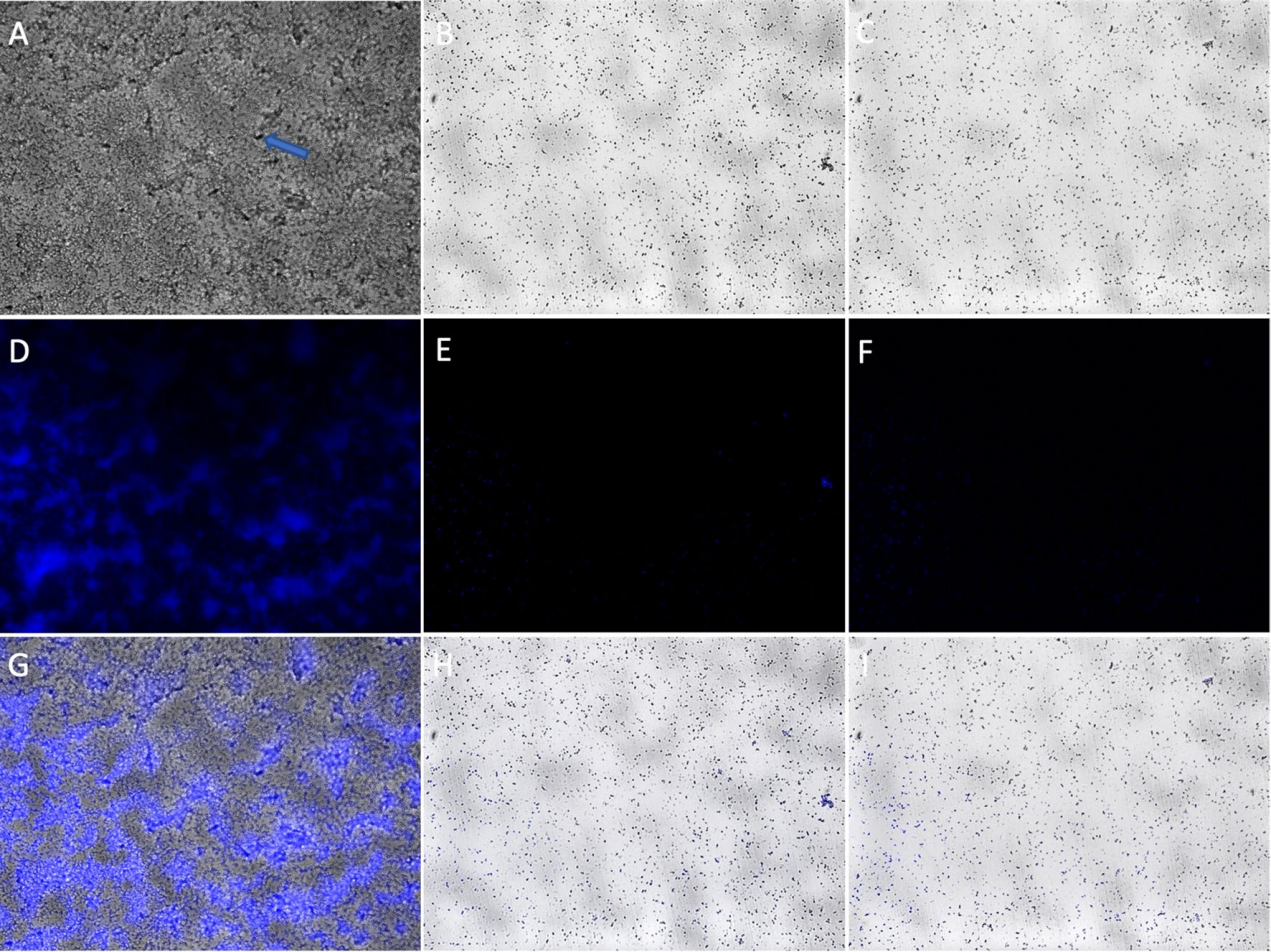
AxioImager A1 light microscope micrographs of *K. pneumoniae* (Kl 315) culture: (**A**,**D**,**G**) after 72 h of incubation without phages; (**B**,**E**,**H**) 24 h of incubation + 48 h of incubation after vB_KpnP_FZ12 treatment; (**C**,**F**,**I**) 24 h of incubation + 48 h of incubation after bacteriophage cocktail treatment; (**A**–**C**) transmitted light micrographs; (**D**–**F**) DAPI fluorescence micrographs; (**G**–**I**) transmitted light + DAPI fluorescence combined micrographs; (**A**) water channel is marked with a blue arrow. Magnification × 400. Experiment was conducted in at least three repetitions; representative slides were presented.

After 72 h of incubation, a mature biofilm with water channels was formed on control glass slides (Fig. 2A,D,G). Only single cells and small colonies were observed on the experimental glasses with the addition of single vB_KpnP_FZ12 (Fig. 2B,E,H) or three-phage cocktail (Fig. 2C,F,I). The antibiofilm activity of the phage cocktail was similar to that of single vB_KpnP_FZ12.

### Scanning electron microscope biofilm imaging

**Figure 3:**
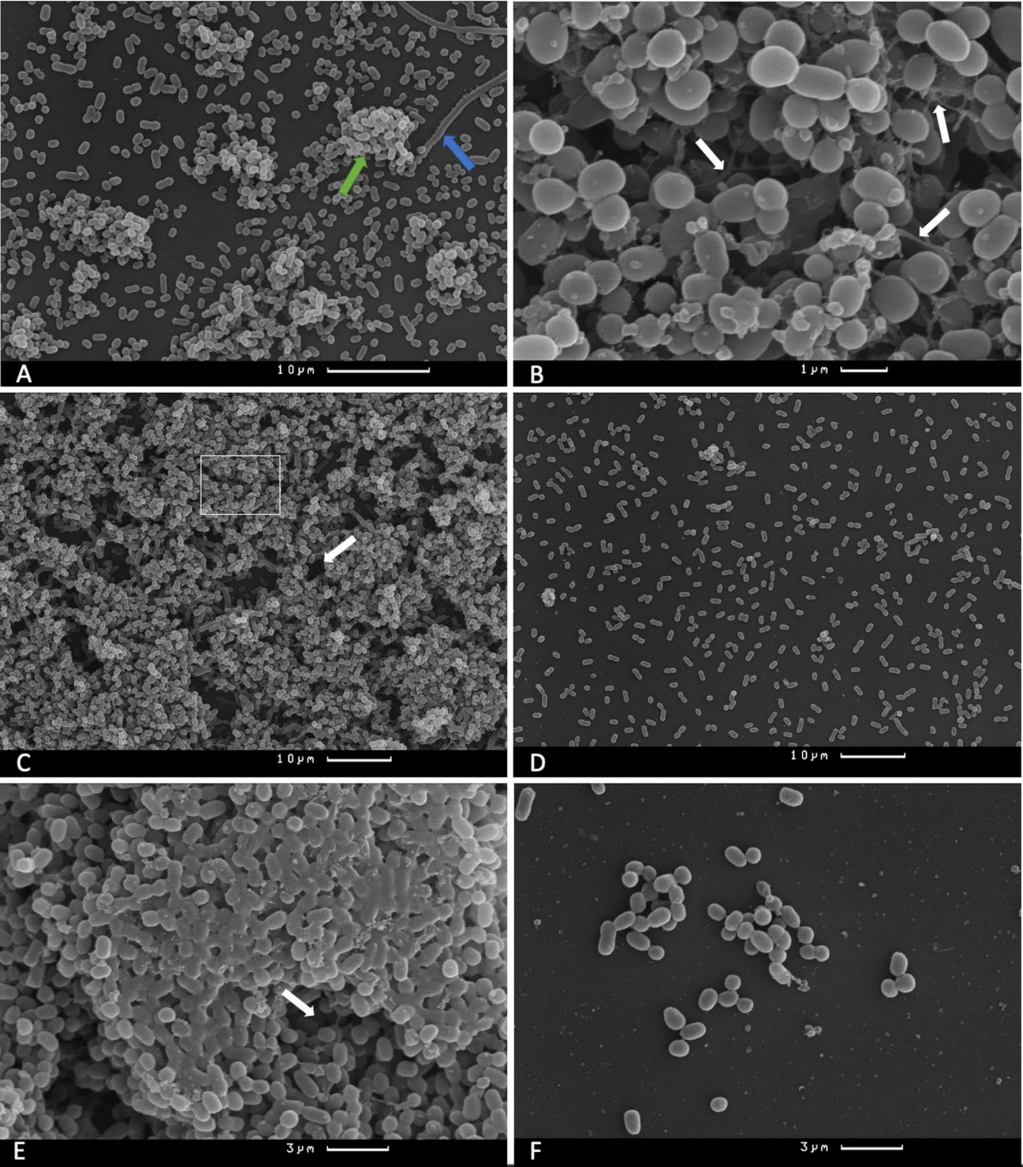
Camscan S2 scanning electron microscope micrographs of *K. pneumoniae* (Kl 315) culture: (**A**) after 24 h of incubation without phages, green arrow marks the formed microcolony, blue arrow marks the *K. pneumoniae* filaments, magnification × 2500; (**B**) after 48 h of incubation without phages, white arrows indicate the exopolysaccharide connections between bacterial cells, magnification × 10,000, the micrograph represent the enlarged white box section of Fig. 3C; (**C**) *K. pneumoniae* (Kl 315) culture after 48 h of incubation without phages, white arrows indicate fluid channels, magnification × 1500; (**D**) *K. pneumoniae* (Kl 315) culture after 24 h of incubation + 24 h after bacteriophage cocktail treatment, magnification × 1500; (**E**) *K. pneumoniae* (Kl 315) culture after 72 h of incubation without phages, white arrows indicate fluid channels, magnification × 4800; (**F**) *K. pneumoniae* (Kl 315) culture after 24 h of incubation + 48 h after bacteriophage cocktail treatment, magnification × 4800. Experiment was conducted in at least three repetitions; representative slides were presented.

After 24 h of incubation (biofilm formation by Kl 315), small microcolonies and formed filaments were observed on the coverslips incubated without bacteriophage cocktail (Fig. 3A). After incubation for additional 24 h the formation of biofilm with clusters and channels was observed in culture without bacteriophage cocktail (Fig. 3B,C). Incubation for 72 h resulted in the formation of a mature biofilm on the surface of coverslips (Fig. 3E). In samples treated by the bacteriophage cocktail after incubation for 24 h and subsequent incubation for 24 and 48 h, only individual bacterial cells and small aggregates were observed (Fig. 3D,F).

**Figure 4:**
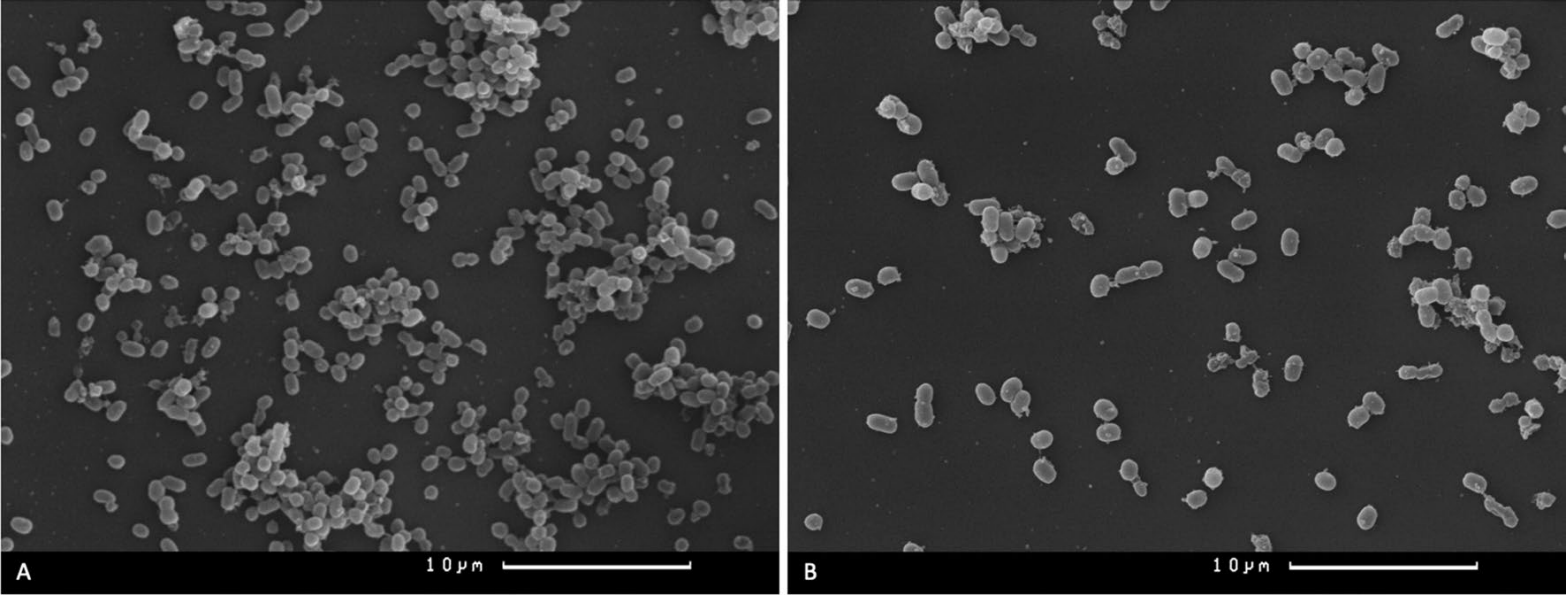
Camscan S2 scanning electron microscope micrographs of *K. pneumoniae* (Kl 315) culture: (**A**) after 24 h of incubation + 24 h after vB_KpnP_FZ12 treatment, magnification × 2800; (**B**) *K. pneumoniae* (Kl 315) culture after 24 h of incubation + 48 h after vB_KpnP_FZ12 treatment, magnification × 2800. Experiment was conducted in at least three repetitions; representative slides were presented.

The results of application of a single vB_KpnP_FZ12 bacteriophage were comparable to those of the bacteriophage cocktail. In samples treated with vB_KpnP_FZ12 alone after incubation for 24 h and subsequent incubations for 24 and 48 h, only single bacterial cells and small aggregates were also observed (Fig. 4A,B).

## Discussion

The previous studies have described various effects of biofilm treatment with bacteriophages. Phage 117 with unconfirmed depolymerase activity (the authors only hypothesized the presence of depolymerases based on the halo obtained on Petri dishes) had no significant effect on biofilm formation when added at the same time as the bacterial culture at MOI of 0.1^20^. Other researchers studied the vB_KpnS_Kp13 phage, which effectively destroyed pre-formed 24 h biofilms in an MOI and time-dependent manner. After 2 h incubation at MOI 10, a loss of 51.8% of biomass was detected. Further, the biomass reduction increased to 54.2%, 57.5% and 72.9% after 12, 24 and 48 h, respectively. These values also decreased at phage-to-bacteria ratios of 1:1 (MOI 1) and 1:10 (MOI 0.1), but still resulted in marked biofilm degradation^21^. The results of its genome analysis showed that the indicated activity was associated with an ORF2-encoded capsule depolymerase. In a study of TSK1 bacteriophage with depolymerase activity researchers compared pre-treatment and post-treatment of biofilms with a single phage. In the pre-treatment experiment TSK1 was added along with the bacterial culture at an MOI of 0.6 and incubated the plate at 37 ± 1 °C for 72 h. In post-treatment experiments, *K. pneumoniae* biofilm of different ages (1–3 days old) was exposed to bacteriophage at 0.6 MOI. It was shown that post-treatment of *K. pneumoniae* biofilms of different ages reduced biomass by 85–100%. Pre-treatment of *K. pneumoniae* biofilms with bacteriophage TSK1 reduced > 99% biomass in the first 24 h of incubation. The biofilm removal tendency was not different for biofilms of different ages as the difference in biomass reduction was not statistically significant^22^. In all conducted experiments, complete eradication of the bacterial culture was not achieved, but a significant decrease in titers and biofilm destruction was observed. However, combined phage-antibiotic therapy may be a solution for complete eradication of bacterial colonization when necessary. A combination of a pre-adapted bacteriophage and antibiotic is known to be used in the treatment of a patient with fracture-related pandrug-resistant *K. pneumoniae* infection^23^. The combination was highly effective in vitro, in 7-day mature biofilms and in suspensions. The researchers observed that ceftazidime/avibactam dose- and time-dependently reduced the number of *K. pneumoniae* bacteria in mature biofilms, but without total elimination. High doses of phage also did not destroy the bacteria in such mature biofilms. However, combinations of high doses of pre-adapted phage M1 and moderate concentrations of ceftazidime/avibactam were significantly more effective than the antibiotic alone. The patient's therapy resulted in objective clinical, microbiological and radiological improvement of the wounds and general condition. In many studies of the effects of bacteriophages on biofilms, no difference in efficacy is observed between the use of a single phage and a cocktail of bacteriophages. For example, a study of the phages 39APmC32, 65APm2833 and 72APm5211 showed that the antibiofilm activity of phage cocktails on 24 h *Proteus mirabilis* biofilms was similar than that of the most active phage^24^. In another study, the pretreatment of 48 h-old biofilm with the *Pseudomonas aeruginosa* phage cocktail resulted in a titer reduction of the same order of magnitude as with a single phage application. However, researchers reported that the application of the phage cocktail rather than single phages prevented the appearance of phage-resistant bacteria^25^. Thus, in the present study we decided to perform a comparison of the efficacy of single phage and phage cocktail in biofilm disruption. The present study demonstrated the ability of both single phage and three-phage cocktail with depolymerase activity to effectively control antibiotic resistant *K. pneumoniae* biofilms. In control of the experiment, all stages of biofilm formation were observed, from single adherent cells, formation of small colonies, clusters, and to biofilm formation and maturation. It is noteworthy that filament formation by *K. pneumoniae* cells was observed in the present study. The production of filaments has been associated with the process of biofilm formation and modern research indicates that filamentation plays a vital role in biofilm development^26^. There is also evidence that exposure to beta-lactam antibiotics is associated with an increased frequency of filament formation^27^, it has been reported that *K. pneumoniae* cells could form filaments in response to cefotiam and cefazolin^28^. We hypothesize that since the biofilm is composed of heterogeneous cells with different metabolism, which plays a key role in adaptation^29,30^, it is possible that exposure to antibiotics leads to the selection of filamentous cells that are already presented in the population.

Bacteriophages were added after 24 h of biofilm development, when such stages of biofilm growth as microcolony and filament formation were observed in the control samples. These time points were chosen based on previous studies^21,22^ and to monitor the effect of bacteriophages on the transition to more mature stages of biofilm formation, as the cocktail could be potentially used in the treatment of different medical devices (including catheters and ventilator tubes) to prevent biofilm formation on them. The addition of bacteriophages to the cell culture prevented biofilm formation; only single cells and small microcolonies were observed in the samples with phages. Application of vB_KpnP_FZ12 phage alone showed the same efficacy in inhibiting biofilm growth as the application of a bacteriophage cocktail composed of three phages (vB_KpnS_FZ10, vB_KpnP_FZ12 and vB_KpnM_FZ14). Complete lysis was not observed, but we presume that this is extremely difficult to achieve with bacteriophage treatment, since even between lytic phages and bacteria more complex mechanisms that regulate lysis exist, such as quorum sensing, hibernation or transient resistance^31–33^. Researchers also observed inherently susceptible but phenotypically antibiotic-resistant subpopulations of bacteria^34^, the presence of persisters in the bacterial population can potentially influence phage infection as well. However, the obtained results show the potential of the selected phage cocktail in the early-stage treatment of different medical devices to prevent biofilm formation on them. Moreover, complete elimination may often not be necessary even in the medical treatment since in humans *Klebsiella spp*. can be found as commensals of the gastrointestinal tract, mouth, and nasopharynx^35^, thus a significant reduction in titers, biofilm disruption, and restoration of normal microbiota balance can become clinically significant. The combined phage-antibiotic therapy could be a solution for the complete eradication of biofilms and bacterial colonization in those cases where it is necessary^23,36^.

The present study shows the potential of the selected bacteriophages in controlling *K. pneumoniae* biofilms. Despite the demonstrated similar efficacy of the single phage and the bacteriophage cocktail in biofilm disruption, phage cocktails are recommended in therapy to prevent phage resistance as well as to improve lytic effects by expanding the phage-host range and increasing the number of target pathogens^37^. We will pursue further studies on the efficacy of bacteriophages vB_KpnS_FZ10, vB_KpnP_FZ12, vB_KpnM_FZ14 in the control of biofilms associated with medical devices and their possible in vivo application to control *K. pneumoniae* biofilm-associated infections.

### Supplementary Information

Supplementary Table 1.

## Data Availability

The complete genome sequences of *Klebsiella pneumoniae* phages vB_KpnS_FZ10, vB_KpnP_FZ12 and vB_KpnM_FZ14 have been deposited in GenBank under the accession numbers MK521904, MK521905 and MK521906, respectively. Raw Illumina reads are available on NCBI SRA under accession numbers SRR10037530, SRR10037529 and SRR10037528, respectively. The associated BioProject accession number is RJNA562287.
