# Supplementary material for: Bacteriophages with depolymerase activity in the control of antibiotic resistant Klebsiella pneumoniae biofilms
Source: Sci Rep. 2023 Sep 13;13:15188. doi: 10.1038/s41598-023-42505-3 (PMC10499987; doi:10.1038/s41598-023-42505-3)
Supplement: Supplementary file 1 — Supplementary Table 1. [file 41598_2023_42505_MOESM1_ESM.pdf]

Bacteriophages with depolymerase activity in the control of antibiotic resistant *Klebsiella pneumoniae* biofilms

Fedor Zurabov*[1,2], Egor Glazunov[1], Tatiana Kochetova[1], Viktoria Uskevich[1], Valentina Popova[1]

[1]Research and Production Center “MicroMir”, LLC, Moscow, Russia
[2]Department of Virology, Lomonosov Moscow State University, Moscow, Russia

Table S1: Antibiotic susceptibility test by disc diffusion method of *K. pneumoniae* strain Kl 315. Interpretations are made according to the EUCAST breakpoint tables. Interpretations marked with * are made according to the CLSI standards because of the absence from the EUKAST standards.

№	Antibiotic	Code	Result, mm	Interpretation	№	Antibiotic	Code	Result, mm	Interpretation
1	AMPICILLIN 10 µg	AMP10	0	R	14	CIPROFLOXACIN 5 µg	CIP5	21	R
2	TETRACYCLINE 30 µg	TE30	22	S	15	RIFAMPICIN 5 µg	RIF5	0	R
3	GENTAMICIN 30 µg	GEN30	19	S	16	NITROFURANTOIN 300 µg	NIT300	13	R*
4	OFLOXACIN 5 µg	OF5	23	S	17	FUSIDIC ACID 10 µg	FC10	0	R
5	ERYTHROMYCIN 15 µg	E15	0	R	18	LINCOMYCIN 15 µg	L15	0	R
6	CHLORAMPHENICOL 30 µg	C30	20	S	19	Tobramycin 10 µg	TOB10	15	R
7	MEROPENEM 10 µg	MRP10	23	S	20	Metronidazole 5 µg	MT5	0	R
8	AZITHROMYCIN 15 µg	AZM15	13	S	21	Moxifloxacin 5 µg	MO5	19	R
9	CEFEPIME 30 µg	CPM30	22	R	24	Amikacin 30 µg	AK30	14	R
10	CEFAZOLIN 30 µg	CZ30	18	R	25	Amoxyclav 30 µg	AMC30	20	S
11	CEFTRIAXONE 30 µg	CTR30	22	R	26	Amoxycillin 30 µg	AMX30	10	R
12	TRIMETHOPRIM 5 µg	TR5	19	S	27	Doxycycline+Hydr 30 µg	DO30	19	S*
13	LEVOFLOXACIN 5 µg	LE5	22	R	28	ERTAPENEM 10 µg	ETP10	24	R
